# Supplementary material for: New Viruses Infecting Hyperthermophilic Bacterium Thermus thermophilus
Source: Viruses. 2024 Sep 3;16(9):1410. doi: 10.3390/v16091410 (PMC11437467; doi:10.3390/v16091410)
Supplement: Supplementary file 1 [file viruses-16-01410-s001.zip › viruses-3175829-supplementary.pdf]

Supplementary data

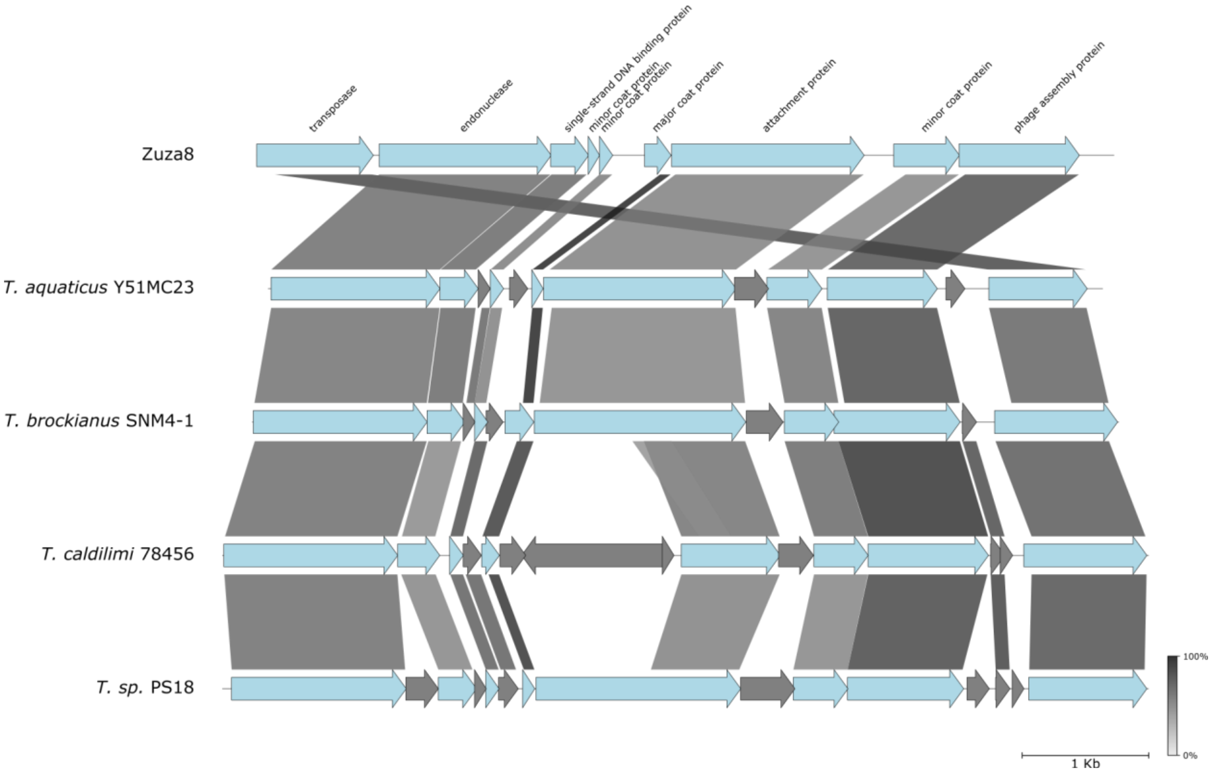

Figure S1. Graphical alignment of the Zuza8 genome with the genomes of *Thermus* isolates. The homologous gene segments shared between Zuza8 and ORFs of the indicated *Thermus* isolates are connected by shading of different degrees of gray based on the amino acid sequence identity levels shown in the bar at bottom right.

```
Zuza8_gp7       MRLAPLLLVVGLLVAGGQSLAADVIDATWRPVVSSATQIALKEPTDIVRVVSGQPARVTV 60
Zuza27_gp7      MPRALLLLVLVLLGVGGPSFAQPQFKDAIKGAVSSLST------PEIIRLPLP-EGVSRP 53
                *  * ****: ** .** *:*   :. : : .*** :        :*:*:     .

Zuza8_gp7       GSAARYVRWGGAVVLGSALVYTALDYFYNVLKAQTGTSLDRWYFWSNPYVEVGRCIDRG- 119
Zuza27_gp7      TTVGRYVRWGGSIILGAALVYTALDWFYNELRRQTSTPLDQWYTGPAPQLA-AYCWNPGW 112
                 :..*******:::**:********:*** *: **.* **:**    * :  . * : *

Zuza8_gp7       ----SYRSWVAVAYVDGRQVNSYAVDNYRECGVPSLRSFTEYWYSLHLVRNYSQLQGVPT 175
Zuza27_gp7      YNGQAYCGWVVVLDSSCEQVGAYSGVV-----GSS--------YE-AILRGY-----VPD 153
                    :*  .**.*   . .**.:*:          *        *.  ::*.*     **

Zuza8_gp7       QYVSPPSGTCPSGWTC----IAVVPQLDRPPLPDWLQSHPDATNGVK-QAVTRYVDSNPI 230
Zuza27_gp7      AVAYTSNGAACGAWSVGSGVPAVLRVGQRQPLPEFLQFHPDAAVALRDQVVAPYIDAHPI 213
                  .   .*:. ..*:      **:   :* ***::** ****: .:: *.*: *:*::**

Zuza8_gp7       GSPSSPYPGVRLEPVPNPNQWTDNPFTRPDIDTDGDGWPDSIEWKEANRTGQNWPDVINN 290
Zuza27_gp7      GSPANPYPGVQLVPVPNPNQWTDNPFTRPDIDTDGDGWPDPVEWREANRRGVPWPDLISN 273
                ***:.*****:* ************************** :**:**** *  ***:*.*

Zuza8_gp7       PTVHPDPNADPDGDGWTNLEELTAGTDPYDASSHPGTRSPTSPWTDTDGDGWPDADEIKL 350
Zuza27_gp7      PEAYPDPNADPDGDGFPNWEEVQKGTDPYDPASRPSPRSPTSPWVDTDGDGYPDRQEIAE 333
                *  .:***********: * **:  ****** :*:*. *******.******:** :**

Zuza8_gp7       GTDPQDPNSHPEG------EPPQKPEEQWPGGPPPGQLKPVDLPEVPQLERRGLPRLDEL 404
Zuza27_gp7      GTDPNDASSHPDPATTPPEQKPEEQPDSWPGGPPPGRIAPVQLPELEVPERERLPEWDKQ 393
                ****:* .***:       : *::  :.********:: **:***:   **. **. *:

Zuza8_gp7       NPVAEAWRTQVVDRVQDRLRNLQDELKNRFPFGIVARLSSGQVQAEGASCSIPVHIGPV- 463
Zuza27_gp7      NPIAEAWKEEVLQRVEQKLGQIRDVLSDRFPFALVLRVGP-RVQTESSPCAFQVNLEGIG 452
                **:****: :*::**:::* :::* *.:****.:* *:.  :**:*.: *:: *::  :

Zuza8_gp7       SSEWNPCSTPFWEAATTFRPIILGLMMVTLTFTLVRRALDVQE        506
Zuza27_gp7      VLNVDICSTPVWQMMAGFRPVLAAGLWLAVGFALLRRSLDVQK        495
                  : : ****.*:  : ***:: . : ::: *:*:**:****:
```

Figure S2. Alignment of amino acid residues of Zuza8 and Zuza27 attachment proteins performed with Clustal Omega.

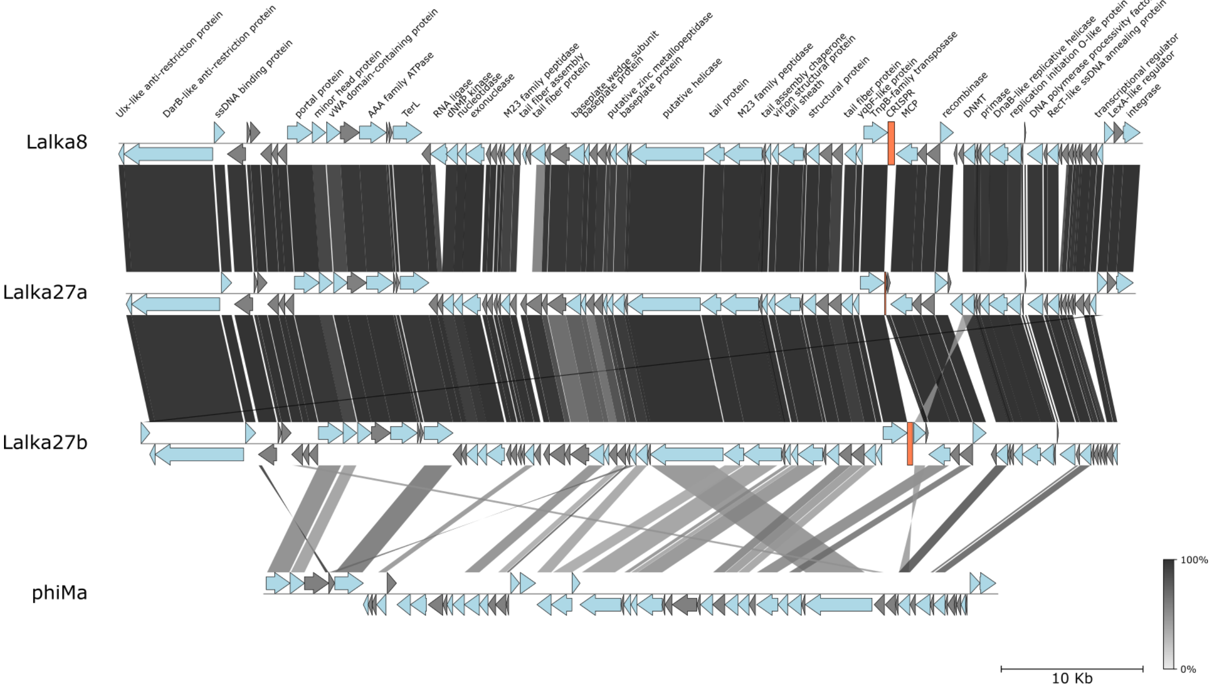

Figure S3. Comparison between the Lalka phages and the phage phiMa genomes. See **Figure 1B** legend for details.

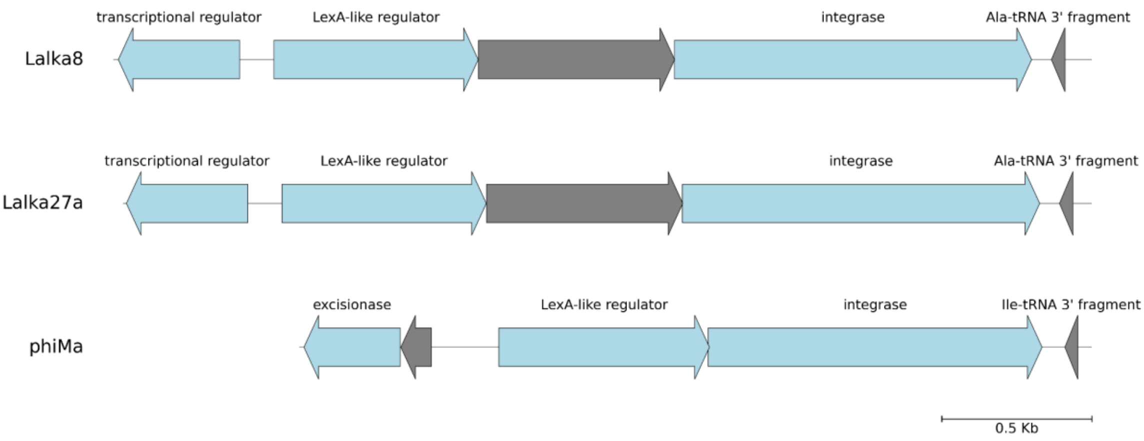

Figure S4. Genomic map of integrase-proximal regions of Lalka8, Lalka27a, and phiMa phages.

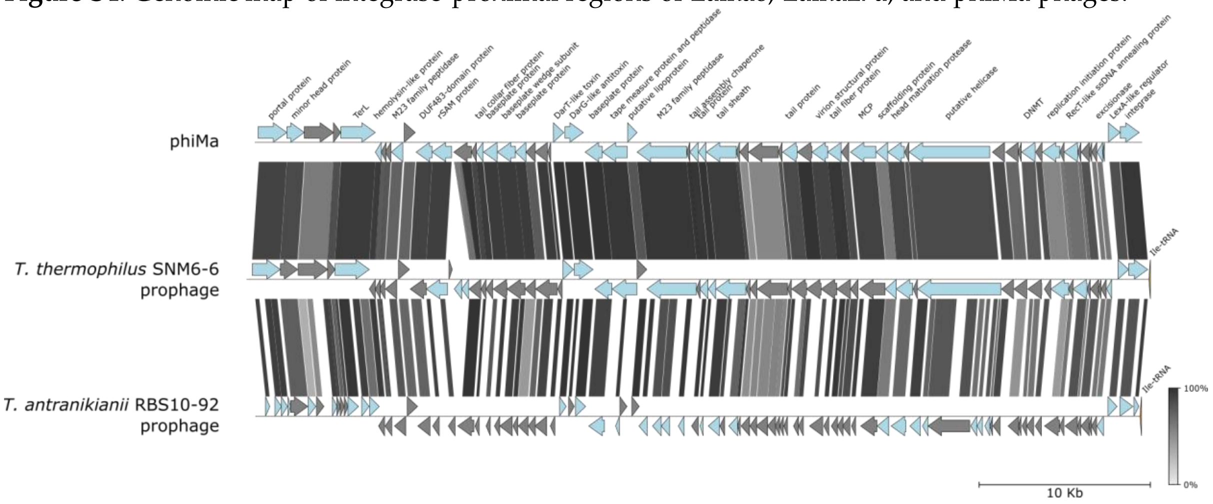

Figure S5. Graphical alignment of the phiMa genome with prophages in *Thermus* isolates.
See **Figure 1B** legend for details. Some ORFs of *T. antranikianii* RBS10-92 are fragmented due to errors generated during assembly from noisy long reads [29].

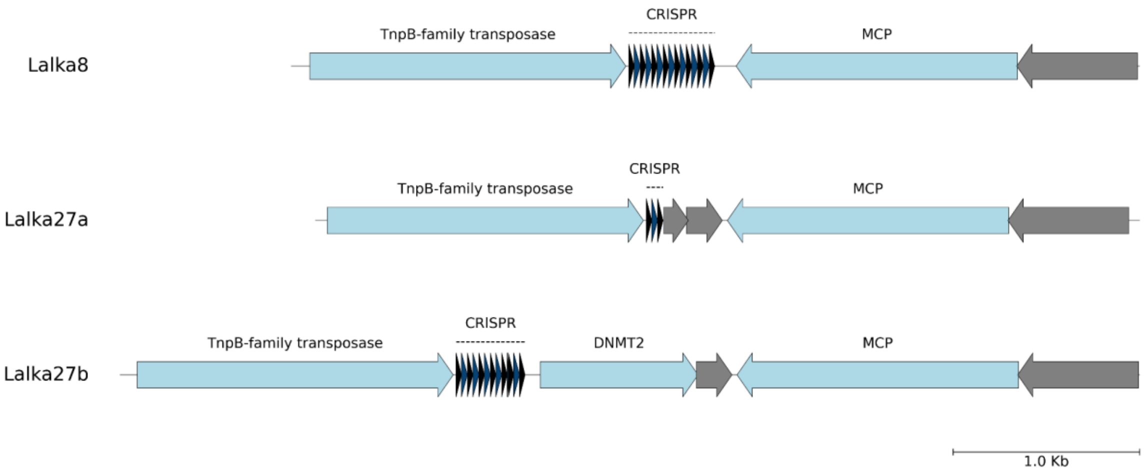

Figure S6. CRISPR-like repeats and the adjacent genes encoded by Lalka8, Lalka27a, and Lalka27b phages. Repeat and spacer units are represented with black and purple triangles.

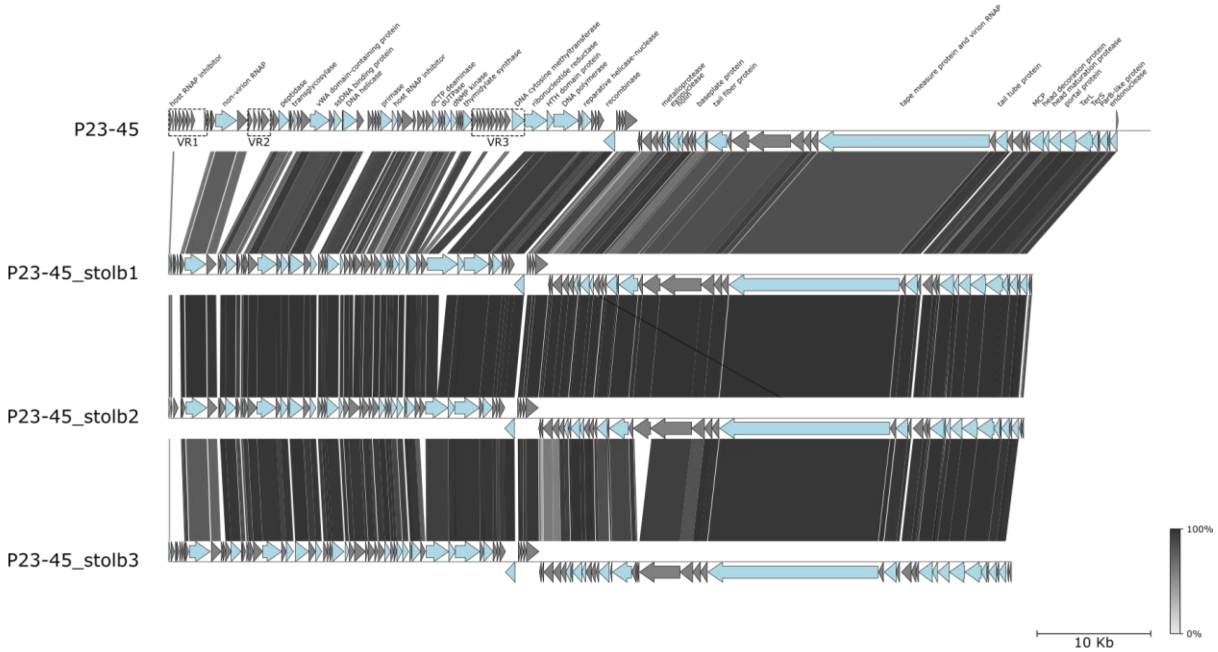

Figure S7. Graphical alignment of the genomes of phage P23-45 and related phages from Stolbovskiy hot springs.

Supplementary table S1. The list of isolated phages, their hosts, and taxonomical groups determined using the tax_myPHAGE tool.

#	Phage	Host	Sample ID	Taxonomy
1	Zuza8	*T. thermophilus* HB8	5	Viruses; Monodnaviria; Loebvirae; Hofneiviricota; Faserviricetes; Tubulavirales; Paulinoviridae; Thomixvirus
2	Zuza27	*T. thermophilus* HB27	6	Viruses; Monodnaviria; Loebvirae; Hofneiviricota; Faserviricetes; Tubulavirales; Paulinoviridae; Thomixvirus
3	Lalka8	*T. thermophilus* HB8	4	Not assigned
4	Lalka27a	*T. thermophilus* HB27	5	Not assigned
5	Lalka27b	*T. thermophilus* HB27	5	Not assigned
6	Tsatsa	*T. thermophilus* HB8	7	Not assigned
7	Cumys	*T. thermophilus* HB8	5	Not assigned
8	Riverbug	*T. thermophilus* HB8	4	Not assigned
9	P23-45_stolb1	*T. thermophilus* HB8	1	Viruses; Duplodnaviria; Heunggongvirae; Uroviricota; Caudoviricetes; Oshimavirus; Oshimavirus
10	P23-45_stolb2	*T. thermophilus* HB8	2	Viruses; Duplodnaviria; Heunggongvirae; Uroviricota; Caudoviricetes; Oshimavirus; Oshimavirus
11	P23-45_stolb3	*T. thermophilus* HB8	3	Viruses; Duplodnaviria; Heunggongvirae; Uroviricota; Caudoviricetes; Oshimavirus; Oshimavirus

Supplementary table S2. Description of phage sampling sites.

Sample ID	Location	Coordinates (lat, lon)	Temperature, °C	pH	Isolated phages
1	Stolbovskiy hot spring, Kunashir	44.006983, 145.683200	71	6.5	P23-45_stolb1
2	Stolbovskiy hot spring, Kunashir	44.007027, 145.683142	70	6.5	P23-45_stolb2
3	Valentina river hot springs, Kunashir	43.9839361,145.6582223	72	6	P23-45_stolb3
4	Valentina river hot springs, Kunashir	43.985114, 145.655747	80	6	Lalka8, Riverbug
5	Valentina river hot springs, Kunashir	43.985097, 145.655873	72	6	Lalka27a, Lalka27b, Zuza8, Cumys
6	Tsaishi hot springs, Georgia	42.426731, 41.799617	80	6	Zuza27
7	Nokalakevi hot springs, Georgia	42.365600, 42.195200	82	6	Tsatsa

Supplementary table S3. Repertoires of defense systems encoded by *T. thermophilus* strains HB8 and HB27.

Defense system	HB8	HB27
CRISPR-Cas system Type I-B	+	+
CRISPR-Cas system Type I-C		+
CRISPR-Cas system Type I-E	+	
CRISPR-Cas system Type III-A	+	+
CRISPR-Cas system Type III-B	+	+
pAgo	+	+
Type II two subunit restriction-modification system	+	-
Type IIG single subunit restriction-modification system	-	+
Hma	+	-
